# The Institutional Library

**Published:** 1913-06-21

**Authors:** 


### THE INSTITUTIONAL LIBRARY.

A NEW SERIES OF POCKET MANUALS.

Essentials of Fever Nursing. By Lytton Maitland, M.D., D.P.H.

Bandaging Made Easy. By M. R. Hosking.

Swedish Exercises. By T. D. Luke, M.D., F.R.C.S.

How to Read and Write Prescriptions. By Lytton Maitland, M.D., D.P.H.

(London : The Scientific Press, Ltd., 28 and 29 Southampton Street, W.C. Price 1s. each net.)

Most attractively got up in serviceable limp covers, and excellently printed and produced, the first volumes of this new practical Pocket-guide Series are bound to create a most favourable impression. To take them in order, Dr. Maitland's "Essentials of Fever Nursing" is a model of what such a condensed text-book on this subject should be. To begin with there are chapters on Infection and on General Management, followed by others in which the peculiar features of each infective fever are dealt with in detail. Throughout the pages the practical note is everywhere sounded; all advice is thoroughly sound and thoroughly comprehensible. The text is always clear, authoritative, and trustworthy; and fever nurses owe a deep debt of gratitude to both author and publisher for this manual issued at such a modest price. The only improvement we would venture to suggest would be the inclusion of an appendix in the next edition, giving the names and addresses of reliable firms in London and the provinces which undertake disinfection of clothing, books, furniture, bedding, and similar domestic equipment which may become subject to actual infection or suspicion of it during a case. Nurses are often asked for such information by patients, and some such list would be of great utility.

Bandaging is one of those arts in which an ounce of practice is worth a ton of theory; and Sister Hosking evidently realises this, because she expressly states that her object is to provide a handy book whereby to recall the various bandages practised at a bandaging class. Her instruction is entirely reliable, explicit, and suited to the needs of beginners; moreover, it is greatly assisted by the ninety figures in the text, which are of the familiar type as seen in larger and more expensive works. If there is one piece of manual dexterity more difficult to learn (or teach) by written rules and diagrams than bandaging is, it must surely be the art of tying knots. Sister Hosking wisely mentions only two or three.

In contrast with bandaging, Swedish exercises are much more capable of exposition by means of diagrams and explanatory letterpress. In Dr. Luke's extremely capable hands the discussion of these remedial and preventive physical exercises was certain to be satisfactory; and so, in fact, it is, for Ling's system is the basis of this, as of the larger and more comprehensive work by the same author. It would be manifestly impossible to compress into 130 pages the whole of that system and its ramifications; but the process of selection and compression has been carried out so judiciously that it is positively astonishing to find how much is included. Those who practise this branch of work as a specialty will probably find this pocket guide, though elementary, yet helpful for their needs; and for the beginner, or the nurse or doctor who but seldom requires to have Swedish exercises carried out, it will prove a most valuable shilingsworth.

Prescription writing in many dispensing practices was rapidly becoming a lost art until the Insurance Act revived it. In Dr. Maitland's handy guide to the subject special features are the comprehensive tables of the doses of drugs in common use, and of the household English equivalents of various preparations which have pharmacopœial Latin names as well. Certainly every medical man ought to know already everything that this guide contains; but certainly many do not, thanks to the pernicious "stock mixture" habit, fomented by the enterprising travellers of manufacturing drug firms. At any rate, there is no excuse for ignorance of the rudiments of prescription writing and posology to continue, since they are carefully expounded in this volume, which is within the reach of the slimmest purse. We shall watch with some interest the further publications of this series, which has made so auspicious a beginning. The standard already reached is a high one, and the further volumes have only to keep it to be as assured of success as those already issued doubtless are.

The Surgery of the Stomach. A Handbook of Diagnosis and Treatment. By Herbert J. Paterson, F.R.C.S. (London : James Nisbet and Co., Ltd., 22 Berners Street, W. 1913. Price 12s. 6d. net.)

Mr. Paterson, whose name in connection with gastric surgery is well known to the profession in this country, was awarded the Jacksonian Prize of the Royal College of Surgeons in 1904 for an essay on "The Diagnosis and Treatment of those Affections of the Stomach which are Amenable to Direct Surgical Interference." The book before us is an expansion of that essay, which takes full cognisance of the newest literature on the subject, and deals in a broad practical way with the essentials of treatment and diagnosis of what may be called "surgical affections" of the stomach. A feature of Mr. Paterson's work is the close attention given to methods of chemical investigation; as the author rightly remarks, "it appears strange that, whereas at the present time bacteriological and pathological examinations are used so extensively in the diagnosis of disease, chemical methods are so rarely made use of in practice and dealt with so briefly in text-books." He insists that the stomach tube should be part of the armamentarium of every practitioner; with this opinion we entirely agree, for the evidence that may be gleaned by a judicious and sane use of the rubber tube is invaluable as an aid to diagnosis in obscure cases. The practitioner would do well to consider carefully Mr. Paterson's *résumé* of clinical and chemical tests; the subject is treated with a commendable conciseness that adds to the merit of the chapters. Apart from this feature of the book, there is little in Mr. Paterson's essay that calls for comment. Perhaps the most important chapter, and the most useful, is that devoted to ulcer of the stomach. Mr. Paterson briefly discusses the indications for operation in cases of gastric and duodenal ulcer; it is worth noting that he recommends a prolonged and thorough course of medical treatment as a necessary preliminary before operation is considered. In the enumeration of the various points to be considered in differentially diagnosing gastric ulcer, he devotes considerable attention to that vague "clinical entity" which is styled "intestinal stasis," but, in our opinion, says far too little about appendicular disease. We certainly do not agree with him that "the pain of appendicular gastralgia is uninfluenced by alkalis," since our own personal experience is directly the opposite. The interaction, if one may call it so, of the nervous plexuses of the stomach and appendix are so intimate most probably, and we know relatively so little about them, that it is as yet impossible to be dogmatic as to final diagnosis; all that can be done is to lay down the general points of difference as a guide to the practitioner in coming to a conclusion.

